# A Rare Skin Lesion Presentation With Sarcoidosis

**DOI:** 10.7759/cureus.67269

**Published:** 2024-08-20

**Authors:** Sayed Hashem, Rafat Harby, Humoud Al-Sabah, Atlal Allafi

**Affiliations:** 1 Dermatology, As'ad Al-Hamad Dermatology Center, Kuwait City, KWT; 2 Dermatopathology, As'ad Al-Hamad Dermatology Center, Kuwait City, KWT

**Keywords:** uncommon skin lesion in sarcoidosis, rare presentation, unusual skin lesion, sarcoidosis, a rare skin lesion presentation with sarcoidosis

## Abstract

Sarcoidosis is a multifaceted systemic disease of uncertain aetiology, pathologically characterised by non-caseating granulomas. Typical symptoms include coughing, dyspnoea, chest pain and lesions affecting the eyes or skin. Cutaneous sarcoidosis frequently accompanies the involvement of other organs, but isolated cutaneous presentations are also observed. We present a case of cutaneous sarcoidosis in a 31-year-old Indian male. The diagnosis of sarcoidosis was confirmed by a skin biopsy, which showed that there is a naked, non-caseating granuloma filling the upper and deep dermis, formed of epithelioid histiocytes and multinucleated giant cells. Treatment with the intralesional steroid triamcinolone acetonide (5 mg/mL) monthly is considered. We hope to raise doctors' awareness of the various forms of sarcoidosis, consequently improving diagnostic skills and patient treatment.

## Introduction

Sarcoidosis is an idiopathic illness that affects several body organs and is characterised by granulomatous inflammation [1]. Roughly 90% of cases involve the lungs, making them the most commonly implicated site [2].

However, cutaneous manifestations are also significant, occurring in 25% of patients with sarcoidosis and presenting as either specific or nonspecific lesions [3,4].

Similar to sarcoidosis in general, cutaneous sarcoidosis is more common in females [3,5]. It is unclear what causes sarcoidosis to develop in the first place. The theory suggests that the immunological response of T helper type 1 (Th1) to one or more extrinsic antigens is dysregulated. This dysregulation could result in the overactivation of inflammatory pathways and the consequent creation of granulomas [6].

## Case presentation

For the case and related photos to be published, we have the consent of the patient.

**Figure 1:**
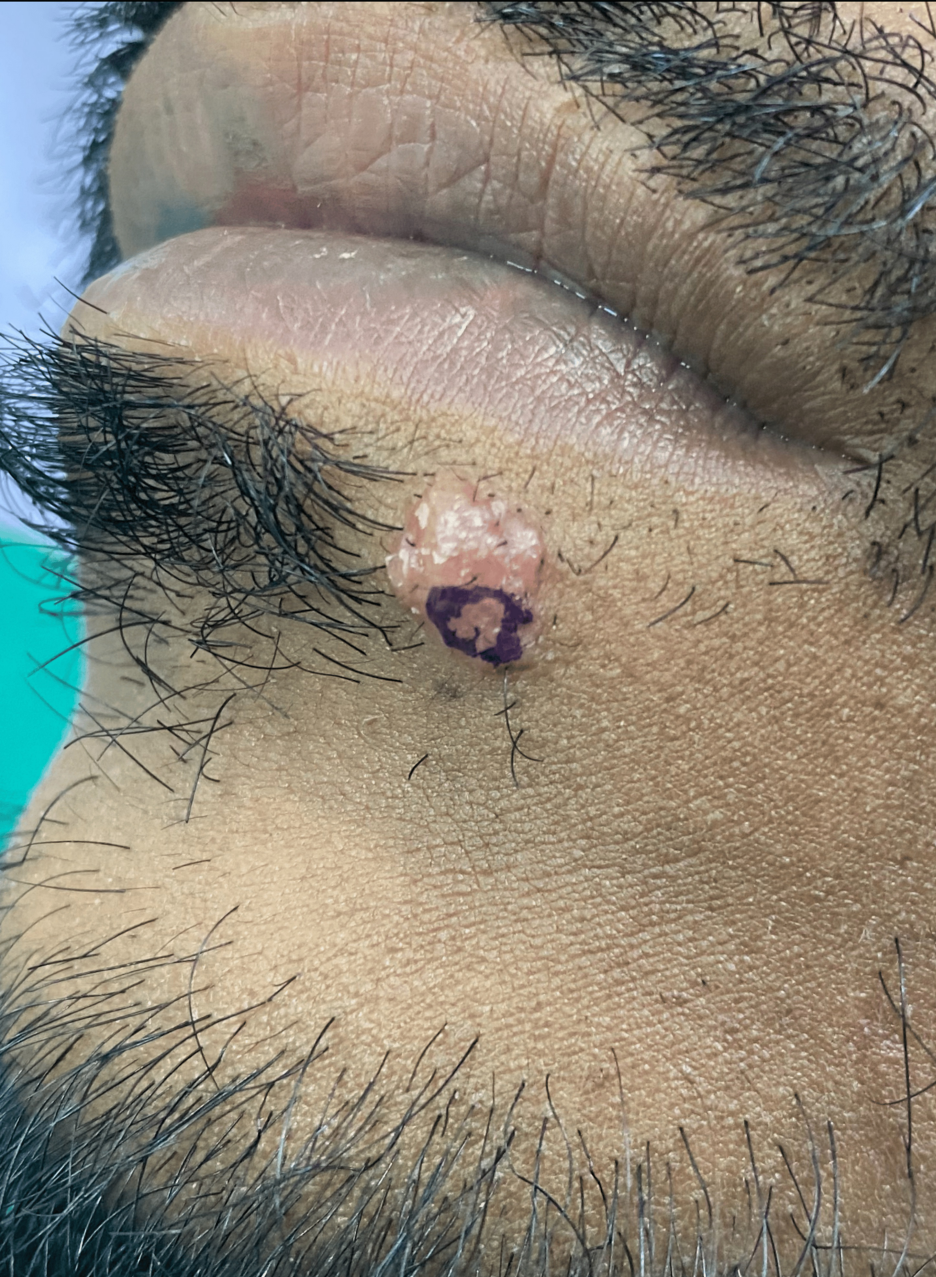
Nodule with a rough pebbly surface on the chin under the left lower vermilion border

A 31-year-old Indian male presented with a two-week history of well-defined, asymptomatic, flesh-coloured nodules with a rough, pebbly surface on the chin, located under the left lower vermilion border (Figure 1). The patient's neurological, respiratory and cardiovascular systems were normal upon additional examination, and no palpable lymphadenopathies or organomegaly was found in the locations that were accessible.

Additionally, the serum angiotensin-converting enzyme (ACE) level was on the upper border (51.6 U/L).

**Figure 2:**
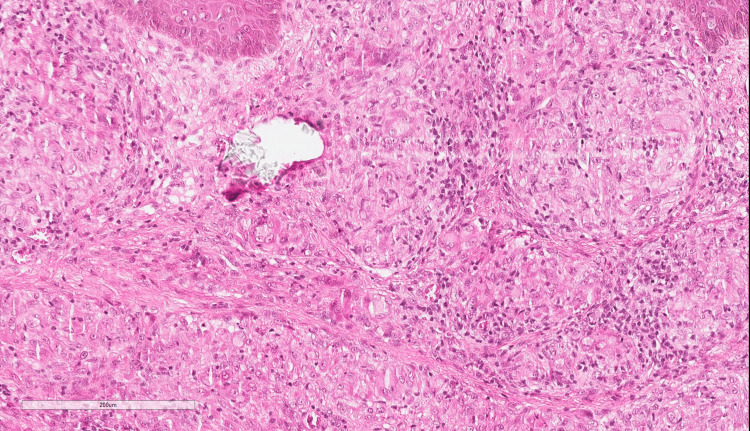
Histopathology of the punch biopsy The histopathological evaluation of the skin biopsy shows an attenuated epidermis. There is a naked, non-caseating granuloma filling the upper and deep dermis, formed of epithelioid histiocytes and multinucleated giant cells. There are few lymphocytic infiltrations. No foreign body was seen

The histopathological evaluation of the skin biopsy shows an attenuated epidermis. There is a naked, non-caseating granuloma filling the upper and deep dermis, formed of epithelioid histiocytes and multinucleated giant cells. There are a few lymphocytic infiltrations. No foreign body was seen (Figure 2).

**Figure 3:**
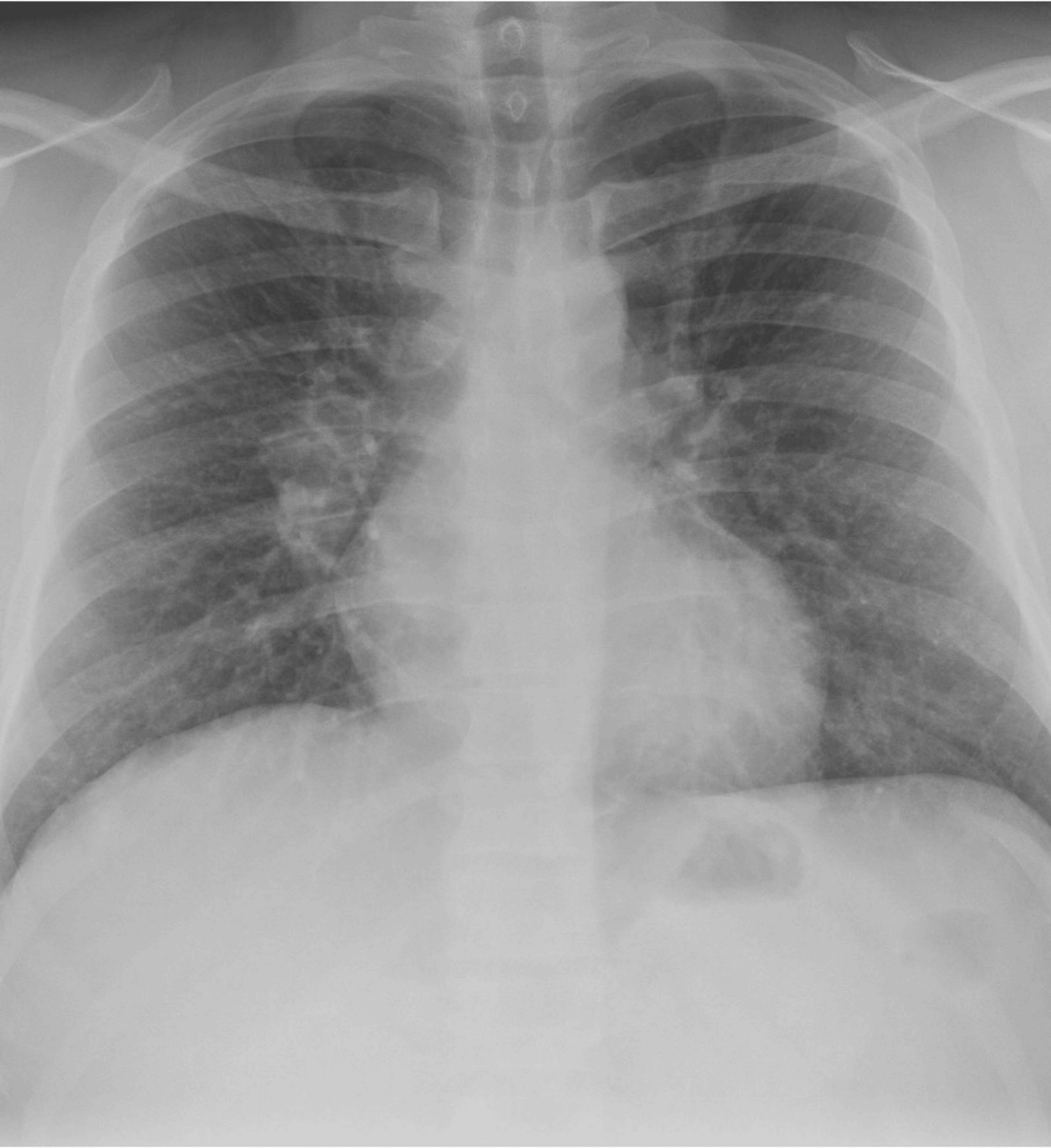
Chest radiograph showing bilateral hilar lymphadenopathy

Notably, the patient's chest X-ray film revealed bilateral hilar lymphadenopathy (BHL) (Figure 3).

For localised cutaneous sarcoidosis, our recommended first-line treatment is intralesional or topical corticosteroid therapy [7].

The treatment plan included receiving intralesional steroid triamcinolone acetonide (5 mg/mL) monthly, as he received two injections in addition to topical steroids and was advised to continue the treatment.

**Figure 4:**
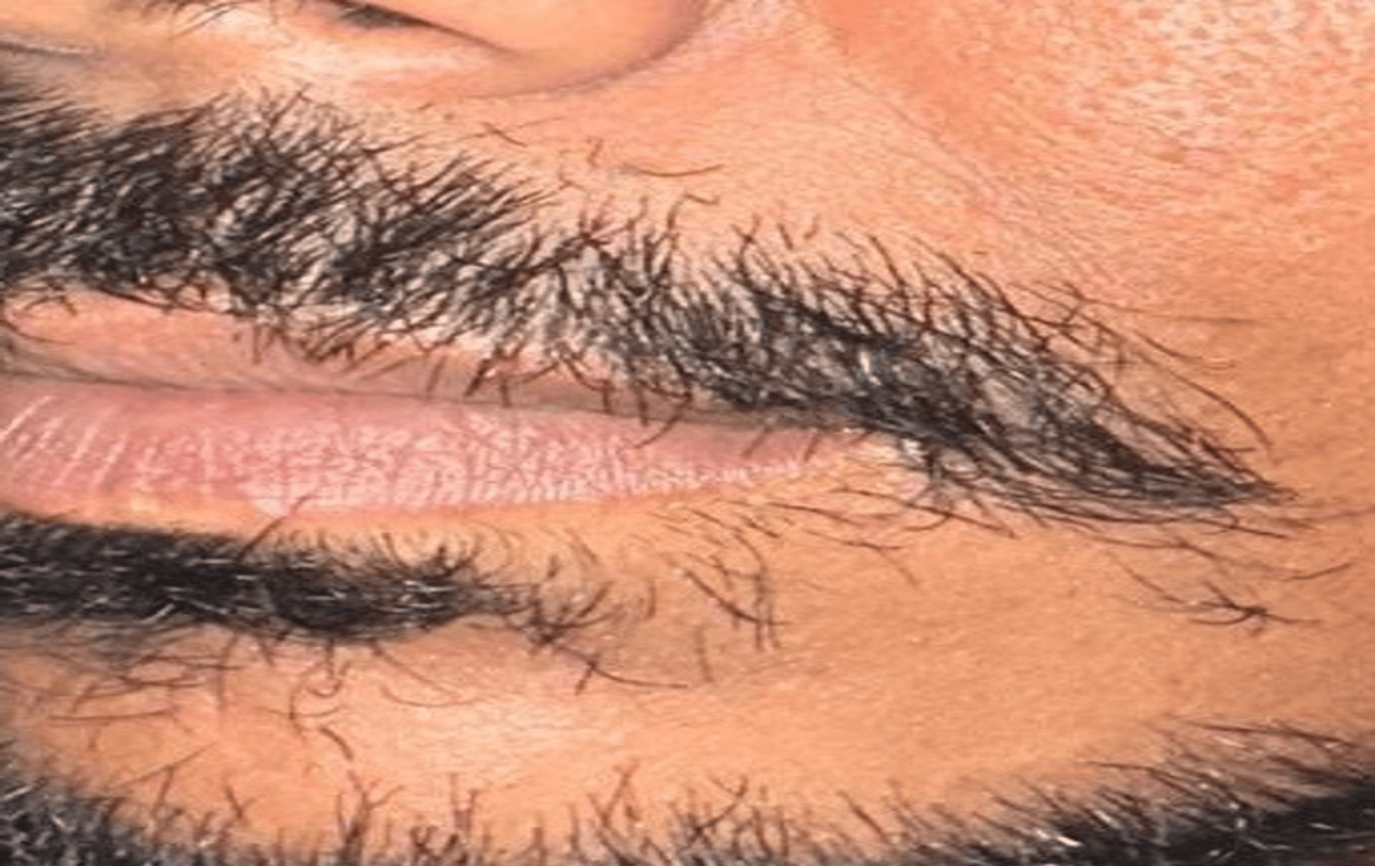
Follow-up after three months After three months, his skin lesions had greatly improved, and the patient was pleased with the result

His skin lesions were significantly improved after three months, and the patient was happy with the outcome (Figure 4).

## Discussion

Sarcoidosis is a multisystem disorder primarily caused by environmental triggers and genetic factors. Pulmonary involvement occurs in approximately 90% of cases, and common cutaneous manifestations include plaques and papules, which are most frequently observed on the trunk, face and upper extremities [3,5].

Sarcoidosis's aetiology is still unknown, yet theories point to a chronic immune response in genetically predisposed people exposed to unknown exogenous antigens [7]. Granuloma formation in sarcoidosis is a hallmark of the disease, involving a complex immune response. It begins with antigen exposure, activates immune cells such as macrophages and dendritic cells and releases cytokines such as interferon gamma (IFN-γ) and tumour necrosis factor-alpha (TNF-α). These cells recruit and activate more macrophages, forming the core of the granuloma, surrounded by lymphocytes and fibroblasts [8]. Clinically, sarcoidosis can present with a wide range of cutaneous symptoms. It can be difficult to diagnose because of its similarities to other conditions, such as erythema nodosum, lupus pernio and maculopapular or plaque-like eruptions; however, research remains in progress in order to clarify this condition [7].

Serum angiotensin-converting enzyme (ACE) levels are elevated in 75% of untreated sarcoidosis patients, but their diagnostic utility is limited due to poor sensitivity and insufficient specificity, with false-positive rates. For an accurate diagnosis, a biopsy of the skin lesion is recommended, and a clinical suspicion should be formed [9].

Topical corticosteroids or intralesional steroids are usually used as standard treatment for cutaneous sarcoidosis. However, systemic steroids may be used with methotrexate or an antimalarial medication if these treatments are ineffective [10]. In our case, we administered the intralesional steroid triamcinolone acetonide (5 mg/mL) monthly.

## Conclusions

This case is notable for the isolated cutaneous presentation of sarcoidosis in a young male patient, which is less expected given the disease's preference for females. It underscores the importance of considering sarcoidosis in the differential diagnosis of atypical skin lesions, even without systemic involvement. In this case, the successful use of intralesional steroids highlights the potential for localised therapy to achieve positive outcomes in similar presentations.
